# Analyzing the influence of gastric intestinal metaplasia on gastric ulcer healing in *Helicobacter pylori*–infected patients without atrophic gastritis

**DOI:** 10.1186/s12876-016-0563-8

**Published:** 2017-01-03

**Authors:** Li-Wei Chen, Liang-Che Chang, Chung-Ching Hua, Bor-Jen Hsieh, Shuo-Wei Chen, Rong-Nan Chien

**Affiliations:** 1 Departments of Gastroenterology and Hepatology, Chang-Gung Memorial Hospital and University, 12F, No 222, Mai-Jin Road, Keelung, Taiwan; 2 Departments of Pathology, Chang-Gung Memorial Hospital and University, 12F, No 222, Mai-Jin Road, Keelung, Taiwan; 3 Departments of Internal Medicine, Chang-Gung Memorial Hospital and University, 12F, No 222, Mai-Jin Road, Keelung, Taiwan

**Keywords:** *Helicobacter pylori*, Intestinal metaplasia, Peptic ulcer

## Abstract

**Background:**

Gastric epithelial hyper-proliferation was reported in patients with *Helicobacter pylori* (*H. pylori*)–infected gastric mucosa with intestinal metaplasia (IM) changes. In patients with gastric ulcer (GU) and IM, the GU may have a different healing rate in comparison to patients without IM. This study aimed to compare the difference in GU healing between *H. pylori–*infected patients with IM and those without IM.

**Methods:**

We retrospectively analyzed patients at the Keelung Chung Gung Memorial Hospital during the period from March 2005 to January 2011. The inclusion criteria were: 1) endoscopic findings of GU and biopsy histological examination plus rapid urease test indicating *H. pylori* infection; 2) gastric IM adjacent to a GU but with no atrophic gastritis changes; 3) patients receiving *H. pylori* eradication triple therapy and 8 weeks of maintenance therapy with a proton pump inhibitor; and 4) patients receiving follow-up endoscopy within the 3^rd^ and the 4^th^ months after treatment.

**Results:**

In total, 327 patients with GU and *H. pylori* infection (136 with IM and 191 without IM) were included. Patients with IM had a higher GU healing rate than those without IM (91.9% vs. 84.3%, *P* = 0.040). Multivariate logistical regression analysis revealed that failure of *H. pylori* eradication (Odds = 4.013, 95% CI: 1.840–8.951, *P* < 0.001) and gastric IM (Odds = 0.369, 95% CI: 0.168–0.812, *P* = 0.013) were the predictors of non-healing GU following treatment.

**Conclusions:**

Patient with gastric IM change may have a higher GU healing rate than those without gastric IM. However, successful *H. pylori* eradication is a more important factor for GU healing than gastric IM.

## Background

Intestinal metaplasia (IM) is a common finding in patients with *Helicobacter pylori* (*H. pylori*) infection. The prevalence of IM in patients with *H. pylori* infection is 30–40% in patients approximately 50 years old [1, 2]. Some studies revealed that the Wingless-Int (Wnt)/β catenin pathway plays an important role in the progression of *H. pylori*-related IM [3–6]. The Wnt signal transduction pathway is also important in intestinal epithelial homeostasis, wound repair and epithelial proliferation [7]. Gastric epithelial hyperproliferation has been observed in patients with gastritis and IM caused by *H. pylori* infection [8–11]. Epithelial cell proliferation is one of the mechanisms governing the repair of a gastric ulcer (GU) [12–14]. Patients with a condition of IM near the GU might have a different outcome of GU healing due to gastric mucosal hyperproliferation. To the best of our knowledge, no study has analyzed whether IM influences GU healing or *H. pylori* eradication. This study aimed to compare the difference in GU healing between *H. pylori–*infected patients with and those without IM adjacent to the GU.

## Methods

We retrospectively analyzed the clinical presentations, endoscopic findings and pathologic records of all patients treated for peptic ulcers at Keelung Chang Gung Memorial Hospital from March 2005 to January 2011. The inclusion criteria in the study were: patients with esophagogastroduodenoscopy (EGD) evidence of active GU in the gastric antrum or body; patients with histological findings by GU biopsy and rapid urease tests indicating *H. pylori* infection with or without IM change; patients who received standard triple therapy (including proton pump inhibitor (PPI), lansoprazole 30 mg or esomerpazole 40 mg, 1 g amoxicillin, and 500 mg clarithromycin twice daily for 7 days) and 8 weeks of maintenance PPI therapy; and patients receiving follow-up EGD and undergoing a rapid urease test and a histological study within the 3^rd^ and the 4^th^ month following treatment. The exclusion criteria were patients receiving PPI or antibiotics two weeks before any of the follow-up EGD studies, and patients taking non-steroid anti-inflammatory drugs (NSAIDs) or aspirin during the healing phase.

If a patient had several EGD studies, only the findings of the 1^st^ and 2^nd^ (follow-up) EGD studies were included in the analysis. The exclusion criteria included patients with underlying malignancy, gastric malignancy revealed by GU biopsy or dysplasia change detected via GU biopsy. In some patients, long-term *H. pylori* infection will induce a progressive gastric atrophy including loss of acid-producing parietal cells. Gastric atrophy leads to lowered gastric acid output which might influence GU healing [15]. Moreover, this study aimed to elucidate the influence of IM adjacent to GU on GU healing and the data of intra-gastric pH could not be available in this retrospective study. Patients with gastric mucosal atrophy according to the results of GU biopsy were also excluded to avoid low gastric acid interfering with GU healing in this study.

### Endoscopic study

Patients who experienced epigastric pain, dyspepsia or acid reflux symptoms received EGD. Wide base ulcer was defined as GU base more than 1.5 cm in size. During the EGD study, GU biopsies (4 specimens from each GU margin mucosa, another specimen from the gastric antrum and one from the incisura angularis of corpus) were obtained except in patients with active ulcer bleeding or NSAID-related shallow ulcers. The rapid urease test (RUT) was administered to confirm the presence of *H. pylori* infection. Patients with positive results from both the histological examination and RUT test were included. In patients who had completed standard triple therapy for *H. pylori* eradication and maintenance PPI therapy, EGD was performed between the 3rd and the 4th month after treatment to evaluate the status of gastric ulcer healing and *H. pylori* eradication success. Therefore, biopsies were repeated for histological analysis and RUT, likewise to the initial EGD. Three stages of GU were defined by endoscopy, based on the cycle of ulcer formation and resolution. Gastric healed ulcer in this study was defined as the regeneration of epithelium that completely covered the floor of the ulcer (scarring status), replacing the white coating ulcer base. Patients with partially healing GU (not scarring status) or active GU detected in the following EGD were recognized as persistent GU in this study.

### Histology and immunohistochemical (IHC) stain for *H. pylori* detection

All patients received GU biopsy for histology (hematoxylin and eosin) and IHC staining (polyclone, Zytomed Systems GmbH, Berlin, Germany) to evaluate *H. pylori* infection status. Histological sections of all biopsies were routinely examined to determine *H. pylori* infection, IM, atrophic gastritis and malignancy. Atrophy of the gastric mucosa was defined as loss of glandular tissue and mucosal thinning changes. IM was detected on the basis of the morphological features in the stomach observed by performing H & E and Alcian blue staining [16–18]. This study applied the most widely used classification, in which there are two types of IM:

1. Complete type IM: presence of small intestinal-type mucosa with goblet cells, a brush border and eosinophilic enterocytes.
2. Incomplete type IM: presence of colonic epithelium with multiple, irregular mucin droplets of variable size in the cytoplasm and absence of a brush border.

IM was scored according to the visual analog scale of the updated Sydney classification [16]. The results of the histological analyses were reviewed by a single experienced pathologist (Dr. Chang LC).

### Rapid urease test (RUT)

RUT (Pronto dry test; Medical Instruments Corporation, Switzerland) was performed. The sensitivity and specificity of RUT for detecting *H. pylori* infection were 99 and 96%, respectively [19].

### Antigen Ki-67 stain for epithelial cell proliferation

For patients with adequate residual biopsy specimens from GU margin after staining with an *H. pylori* antibody, an additional Ki-67 IHC stain was applied to assess cell proliferation. Antigen Ki-67 IHC staining was performed using DAKO autostain agent (Cytomation, Carpinteria, CA). REAL EnVision Detection System, Peroxidase/diaminobenzidine (DAB) (K5007, DAKO) was used to visualize the staining.

### Digital data analysis

**Fig. 1:**
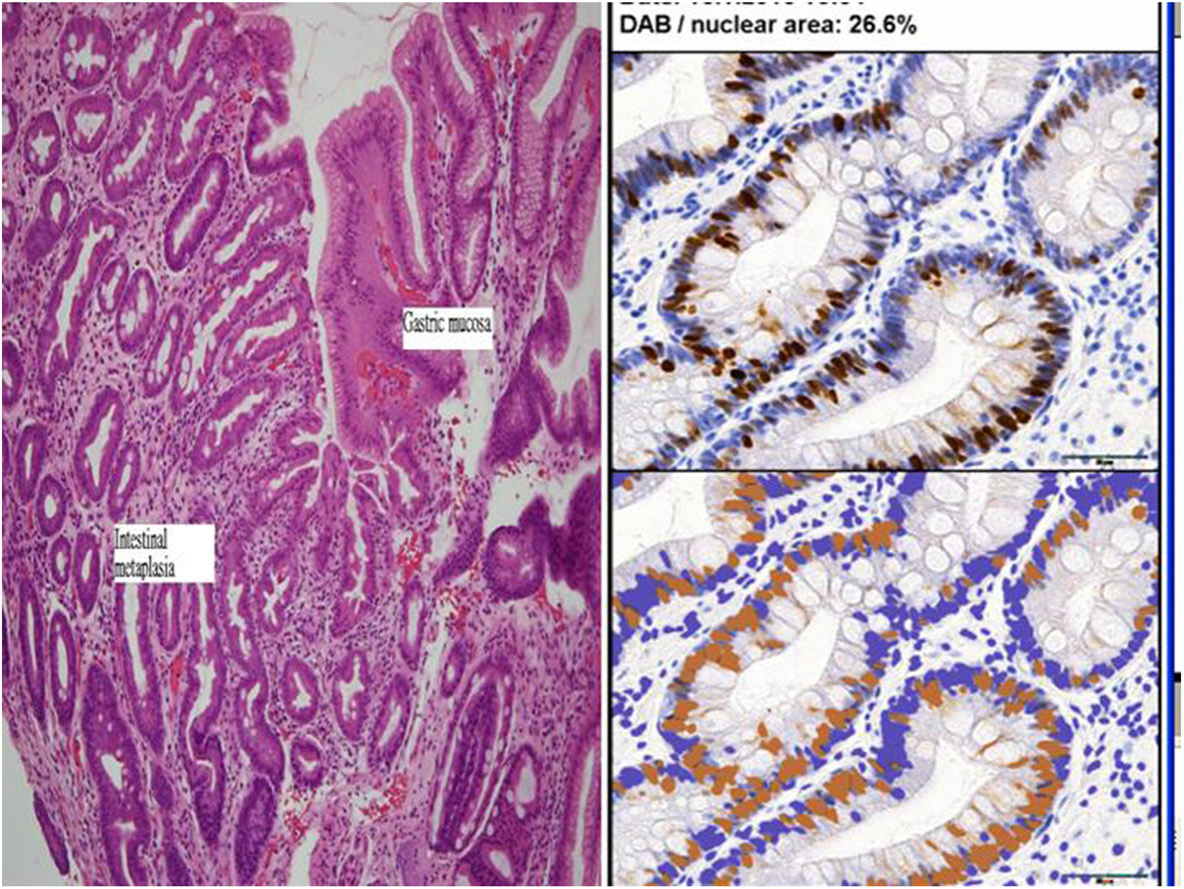
*Left* side: Photomicrographs of gastric intestinal metaplasia. Gastric mucosa on the left show intestinal metaplasia with histological features similar to the small bowel epithelium. Gastric mucosa lined by foveolar cells at the right upper portion. (Hematoxylin and eosin stain; 100×). *Right* side: ImmunoRatio was applied to calculate the percentage of positively stained nuclear area (labeling index, DAB: diaminobenzidine area) among the background hematoxylin stain area (Ki-67 index: DAB/nuclear area = 26.6% in this slide)

Digital data analysis was performed with computer software to prevent manual or inter-observer bias for the Ki-67 index score counting. The digital data analysis was processed with ImageJ (1.45i) [20]. The color deconvolution plug-in was used to separate the stains into two 8-bit component images: the DAB image and the hematoxylin image. The DAB image was used for density measurement. We calculated the percentage of positively stained nuclei (labeling index) by using a color deconvolution algorithm to separate the staining components and an adaptive threshold for nuclear area segmentation (Fig. 1, right side) [21]. Five pictures from the targeted gastric epithelium adjacent to GU were applied for digital data analysis. The result was recorded as a mean Ki-67 labeling index (%).

**Fig. 2:**
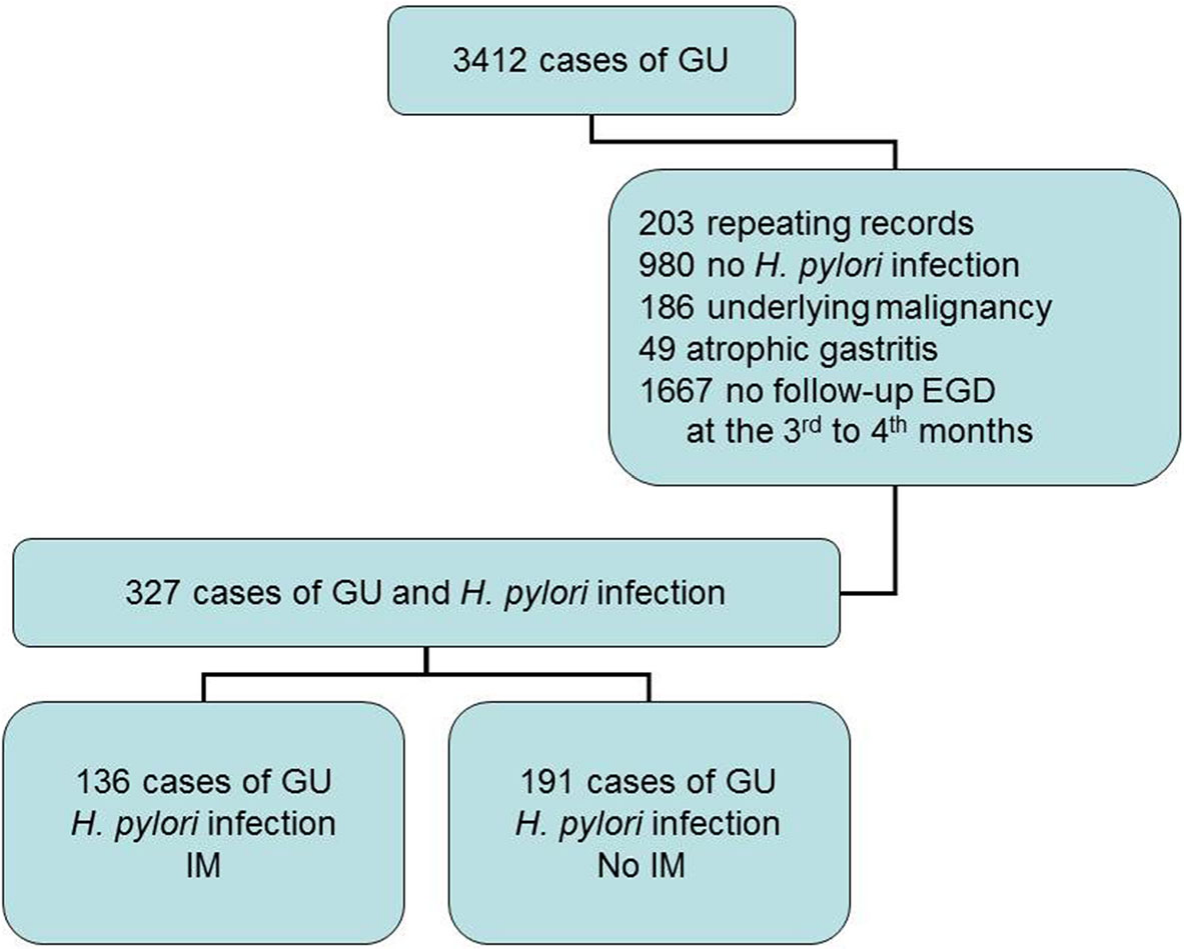
Case selection flow chart. GU: gastric ulcer; EGD: esophagogastroduodenoscopy; IM: intestinal metaplasia

### Statistical methods

To the best of our knowledge, there are no previous reports on peptic ulcer-healing rate or *H. pylori* eradication rate following PPI administration in patients with GU and IM. Because GU patients with *H. pylori* infection and IM exhibit gastric epithelial hyperproliferation, the null hypothesis was that patients with IM had a higher rate of GU scarring (prediction: 95%) than patients without IM (80% in our previous study) following standard triple eradication and PPI therapy [18]. To obtain a power of 0.8 at the significant level of 0.05, the minimal required number of patients was 70 for each group. Hence, it was reasonable to enroll at least 140 patients in this study.

Continuous data were expressed as the mean ± standard deviation (SD). Continuous data were evaluated using the paired-*t* test if the sample size was more than 30 in each group and the Mann–Whitney test was applied if the sample size was less than 30. The chi-square test was used for nominal data. Categorical data were analyzed with the chi-square test or Fisher’s exact test, where appropriate. All statistical tests were 2-tailed. A *P-*value of <0.05 was considered statistically significant. The correlation coefficients such as Pearson’s correlation coefficient, Point bi-serial correlation coefficient and Spearman’s co-efficiency rho were appropriately chosen based on the data types including numerical, nominal or ordinal data. A multivariate logistic regression analysis was applied for the predictors’ evaluation for non-healing GU and persistent *H. pylori* infection after treatment.

Statistical analyses were performed using the Statistical Package for the Social Science software version 16.0 for Windows (SPSS, Chicago, IL, USA).

This research was approved by the Institutional Review Board of the Chang-Gung Memorial Hospital (IRB No: 99-2661B). Written informed consent was obtained from all enrolled subjects in this study.

## Results

We initially included 3412 cases of GU, which were diagnosed by EGD and pathologic studies. In total, 136 cases of GU with IM (IM group), and 191 cases of GU without IM (no IM group) were enrolled in the final analysis (Fig. 2). Most patients with gastric IM were of the incomplete type (122/136, 89.7%). Because patients with atrophic mucosa in histological findings were excluded in this study, the majority of patients had minimal foci of IM and were scored as 0 (absent) or 1 (mild) (107/136, 78.7%) by using the Sydney system visual analog scale. Only 29 patients with incomplete IM were scored as 2 (moderate). No patient was scored as 3 (marked).

Demographic and other characteristics of the patients are listed in Table 1. No significant inter-group differences were observed in the distribution of gender, symptoms, personal history, co-morbidities, GU size and location. Patients in the IM group were older than those in the no IM group (mean age, 63.5 ± 12.3 years vs. 59.4 ± 13.4 years, *P* = 0.004). Patients with IM had higher GU-healing rate than those without IM (92.1% vs. 84.3%, *P* = 0.036). However, there were no significant differences in the *H. pylori* eradication rates between the two groups [81.6% (IM group) vs. 85.3% (no IM group), *P* = 0.405]. A subgroup analysis from 274 patients with successful *H. pylori* eradication was performed and the result revealed no significant difference in GU healing rate between patients with IM (104/111, 93.7%) and those without IM (144/163, 88.3%) (*P* = 0.125).

**Table 1:** Demographics and other characteristics of patients with gastric ulcer and *H. pylori* infections

Characteristic	IM	No IM	*P* value
Number	136	191	
Age (years)*	63.3 ± 12.1	59. 3 ± 13.4	0.006
Gender (F/M)	58/78	74/117	0.478
Symptoms			
Abdominal pain	100 (73.5%)	149 (78.0%)	0.349
Abdominal fullness	54 (39.7%)	82 (42.9%)	0.560
GERD	14 (10.3%)	17 (8.9%)	0.672
Personal history			
Smoking	32 (23.5%)	49 (25.7%)	0.661
Alcohol	24 (17.6%)	32 (16.8%)	0.833
Co morbidity			
Cirrhosis	6 (4.4%)	11 (5.8%)	0.589
Uremia	5 (3.7%)	7 (3.7%)	0.996
Diabetic mellitus	28 (20.6%)	34 (17.8%)	0.526
IM type			
Complete type	14 (10.3%)		
Incomplete type	122 (89.7%)		
Gastric ulcer			
Wide base (>1.5 cm)	23 (16.9%)	21 (11.0%)	0.122
Location			
antrum	62 (45.6%)	101 (52.9%)	0.194
body	74 (54.4%)	90 (47.1%)	0.194
GU-scarring rate	125 (91.9%)	161 (84.3%)	0.040
*H. pylori* eradication rate	111 (81.6%)	163 (85.3%)	0.368

*Data presented as the mean ± standard deviation; number (%)

*IM* Intestinal metaplasia, *GERD* Gastro-esophageal reflux disease, *GU-scaring rate* Healed (scarred) gastric ulcer rate (%) following treatment, *H. pylori eradication rate* *H. pylori * eradication success rate following treatment

The bivariate correlation test revealed that non-healing GU (NHGU) was positively correlated with age and persistent *H. pylori* infection (failure of *H. pylori* eradication), but negatively correlated with IM (Table 2). Because patients with IM were older than those without IM in this study, multivariate logistical regression analysis was performed to determine the predictor of GU healing by adjusting the factor of age (Table 3). As positive predictors for NHGU, the analysis revealed age [odds ratio (OR) =1.035, 95% confidence interval (CI) = 1.007–1.064; *P* = 0.015] and persistent *H. pylori* infection [OR = 3.924, 95% CI = 1.857–8.294, *P* < 0.001]. A negative predictor was IM [OR = 0.366, 95% CI = 0.170–0.792, *P* = 0.011].

**Table 2:** A correlation analysis among factors of non-healing gastric ulcer, persistent *H. pylori *infection and other factors, data are presented as correlation coefficients (Spearmen’s rho)

Variables	NHGU	Persistent Hp
Gender	0.010	0.024
Age	0.126^*^	0.069
Smoking	−0.025	−0.002
Alcohol	−0.025	−0.046
Cirrhosis	0.078	0.084
Uremia	0.073	−0.042
DM	0.029	0.020
Wide base ulcer	0.013	0.118^*^
Persistent Hp	0.209^*^	1.000
IM	−0.113^*^	0.050
NHGU	1.000	0.209^*^

*DM* diabetic mellitus, *Wide base ulcer* gastric ulcer base more than 1.5 cm, *IM* intestinal metaplasia besides the gastric ulcer, *Persistent Hp* persistent *H. pylori * infection after treatment (eradication failure), *NHGU* non-healing gastric ulcer after treatment

^*^ *P*-value < 0.05

**Table 3:** Predictors for non-healing gastric ulcer after treatment according to logistic regression analysis

Variables	Odds ratio (95% CI)	*P-*value
Age	1.035 (1.007–1.064)	0.015
Persistent Hp	3.924 (1.857–8.294)	<0.001
IM	0.366 (0.170–0.792)	0.011

*CI* confidence interval

For failure of *H. pylori* eradication, only the factor of NHGU was a predictor. IM was not a predictor and was not correlated with failure of *H. pylori* eradication (Table 4).

**Table 4:** Predictors for persistent *H. pylori* infection after treatment according to logistic regression analysis

Variables	Odds ratio (95% CI)	*P-*value
Wide base ulcer	2.256 (1.053–4.836)	0.036
NHGU	3.808 (1.836–7.896)	<0.001

Because this was a retrospective analysis, only adequate GU biopsy specimens from patients before and after *H. pylori* eradication therapy were used for the IHC stain study. A total of 64 GU biopsy specimens (30 from patients with non-IM, 34 patients with IM) underwent Ki-67 staining (Table 5). There was no significant difference in Ki-67 index between patients with IM and those without IM. The mean Ki-67 index change before or after treatment was only marginally different between patients with IM and those without IM (0.5 ± 1.7 vs. 0.6 ± 1.9, *P* = 0.06).

**Table 5:** Mean Ki-67 stain index in patients before and after *H. pylori* eradication and maintenance of proton pump inhibitor therapy for 8 weeks

Ki-67 index	No Intestinal metaplasia (*n* = 30)			Intestinal metaplasia (*n* = 34)		
	Before	After	*P* value	Before	After	*P* value
Mean index (%)	19.1 ± 15.0	17.3 ± 15.6	0.20	19.8 ± 13.4	17.8 ± 12.2	0.34
Change (%)*	0.6 ± 1.9			0.5 ± 1.7		0.06

*Index change (%) calculated as the mean Ki-67 index (before-after)/before × 100%

## Discussion

IM is a common pathologic finding in patients with endoscopy-proven GU [22–24]. Previous studies focused on the precancerous condition of IM; however, few studies have investigated the influence of IM on gastric ulcer healing and *H. pylori* eradication. Factors such as age, smoking and *H. pylori* eradication were reported as important factors for GU healing [25–27]. In our study, although patients with gastric mucosal IM had a higher GU healing rate than those without IM, the GU healing rates were not significantly different when the focus was on the patients with successful *H. pylori* eradication. Multivariate logistic regression analyses revealed that failure of *H. pylori* eradication was a positive predictor (OR = 4.013) and IM was a negative predictor (OR = 0.369) for non-healing GU. Hence, successful *H. pylori* eradication is a more important predictor than IM for GU healing in this study.

The mechanisms associated with GU healing and repair include epithelial cell proliferation, growth control, epidermal growth factors, angiogenesis and inhibition of acid secretion [12, 13, 28, 29]. Although our original hypothesis was that cell hyperproliferation in IM might influence GU healing, we found that there was no significant difference in cell proliferation Ki-67 stain index between the IM group and the no IM group. Antigen Ki-67 is a nuclear protein that is associated with cellular proliferation and ribosomal RNA transcription. The Ki-67 protein is present during all active phases of the cell cycle (G1, S, G2 and mitosis), but is absent from resting cells (G0). In previous studies, patients with *H. pylori* infection and chronic gastritis exhibited significantly increased rates of epithelial cell proliferation. Cell proliferation decreases after successful *H. pylori* eradication [30, 31]. Although the mean Ki-67 index was decreased (decreased cell proliferation) following *H. pylori* eradication in our study, there was no significant difference in mean Ki-67 index between patients with IM and those without IM. The condition of gastric mucosal cell proliferation might be due to the status of *H. pylori* infection and chronic gastritis, but not the status of IM [30, 31].

There were two limitations in the present study. First, this study only included patients with GU and IM adjacent to GU, but patients with atrophic gastritis were excluded. It is common to detect atrophic gastritis and IM coexistence in patients with an *H. pylori* infection [16–18]. Although the intra-gastric juice pH value and serum pepsinogen value are important for the evaluation of atrophic gastritis and GU healing, these data were not available in this retrospective study. It would be difficult to elucidate whether atrophic gastritis or IM has an influence on GU healing when a patient has both pathologic findings of atrophic gastritis and IM in the stomach. Hence, we excluded the patients with atrophic gastritis detected via histological examination in this study. Second, most of the IM types in this study were mild according to the Sydney system. We could not compare differences in GU healing between patients with mild forms of IM and those with marked forms of IM.

## Conclusions

Patients with gastric IM may have a higher GU healing rate than those without gastric IM. Age, successful *H. pylori* eradication and gastric IM were the predictors for GU healing. However, successful *H. pylori* eradication was a more important factor for GU healing than gastric IM.

## Abbreviations

*H. pylori*: *Helicobacter pylori*
GU: Gastric ulcer
IM: Intestinal metaplasia
EGD: Esophagogastroduodenoscopy
PPI: Proton pump inhibitor
RUT: Rapid urease test
DAB: Diaminobenzidine
IHC: Immunohistochemical
SD: Standard deviation
